# Determinants of Electricity Consumption Intensity in China: Analysis of Cities at Subprovince and Prefecture Levels in 2009

**DOI:** 10.1100/2012/496341

**Published:** 2012-08-13

**Authors:** X. H. Xia, Yi Hu

**Affiliations:** ^1^Institute of China's Economic Reform and Development, Renmin University of China, Beijing 100872, China; ^2^Academy of Mathematics and Systems Science, Chinese Academy of Sciences, Beijing 100190, China

## Abstract

China has experienced great social and economic vicissitudes that caused the vast complexity and uncertainty for electricity consumption. This paper attempts to identify the main determinants of the electricity consumption intensity by using the data from Chinese cities at subprovince and prefecture levels in 2009. The key category factors, including urban morphology, industrial structure, regulation context, urbanization degree, price, natural condition, and resource endowment, are abstracted and the influence of these determinants is evaluated by adopting the finite mixture models. The variation of each determinant across regions, the comparative weights of all the factors, and the detailed classifications of the cities are reported for facilitating the understanding of electricity consumption in China. The corresponding policies for electricity administration are addressed as well.

## 1. Introduction

Industrialization and urbanization are two distinguishing features in the present social and economic development of China. The rural-to-urban migration and changes in industrial and economic structure are regarded as two major factors which determine the electricity consumption and economic output. The cities at subprovince and prefecture levels especially districts under the jurisdiction of cities which are, in general, cores of the region, play the leading roles and have the dominant positions in the processes of social development.

In the hierarchy of the administrative divisions of China mainland, cities at subprovince and prefecture levels (hereinafter briefly called the cities) cover 47.859% of China's total land area, 87.260% of total population, and 93.903% of Gross Regional Product (GRP), while 52.847% of GRP, 49.843% of total investment in fixed assets, and 51.695% of local government revenue are concentrated in the districts under the jurisdiction of the cities with only 12.476% of population and 27.654% of land area of the cities in 2009 [1, 2]. Meanwhile, the electricity consumption of the districts of the cities amounts to 1639.546 billion kwh which is about 44.273% of the total electricity consumption in China [2].

Prefecture level divisions (including subprovincial level, similarly hereinafter) are the second level of the administrative structure in the Constitution of China. The government at this level undertakes multiple responsibilities in terms of the administration of energy, economy, and environment issues.

The progress of urbanization and industrialization is always highly promoted by local governments, as it could bring about economic growth and better official performances which are two of the most important measures of GRP. Under the system of intergovernmental fiscal decentralization, about 52.400% of fiscal revenues are extracted by central government in 2009 [1]. The fiscal revenue of local government is another incentive to contest for output increase between different administrative divisions such as counties. As a general rule, the government at higher level is entitled to balance the development of the jurisdictional area. Hence the government at prefecture level is not only the implementer and promoter of urbanization and industrialization, but also the coordinator and participator of regional competition.

As a basic decision-making unit, the government at prefecture level is responsible for designing the layout of industry, especially that of small- and medium-sized enterprises (SMEs). For instance, the industrial park, being regarded as the incubator of local development, is often planned by government at this level. Meanwhile they also own the right to select industrial projects. In fact, the local government could reject the industrial settlement even programmed by the higher authorities for environmental or other reasons, or strive for it in order to earn more fiscal revenues when the projects are not settled. Thus the spatial arrangement of urban morphology and economy is mainly influenced and managed by authority at this level.

It is also a crucial node for the execution of energy policies including the regulation of electricity consumption in consideration of the more direct contact with local enterprises compared with higher authorities. The Chinese energy systems are intensively studied from the perspectives of international contexts [3, 4], provinces and regions [5, 6], macro systems [7–11], historical evolution [12, 13], and economic sectors [14, 15]. Further study focusing on the analysis of divisions at the prefectural level could provide detailed information on the enforcement of the energy legislation, the motivation for the behavior of local government, and the heterogeneity and exogenous variables determining the electricity consumption.

As an efficient method allowing introducing unobservable heterogeneity, which exists largely in Chinese regions due to the unbalance of development in terms of electricity consumption, the finite mixture model has been widely used for capturing the different effects among variables in regressions and segregating samples for the convenience of analysis. The application of finite mixture models includes Beard et al. [16] for the identification of the technical diffusion and changing among US banks, Wedel and Desarbo [17] for the clustering of market segment on the satisfaction study in Europe, Yau et al. [18] for the estimation of the effects of pertinent factors which influenced the hospital neonatal length of stay in Austria, and Imai and Tingley [19] for the measuring the performance of rival theories. Considering the possibility of inaccurateness caused by statistical mistakes of migration population and the consistency with China's statistical system, the electricity intensity defined as the ratio of electricity to GRP is used as the indicator of electricity consumption in different areas. This paper aims to abstract the key factors determining the electricity consumption intensity of the cities at prefecture level in China, evaluate the influence of the processes of urbanization and industrialization on the electricity consumption intensity, compare the difference of these determinants' impact, and understand the channels through which each variable functions for policy makers.

## 2. Literature Review

The determinants of electricity consumption are extensively studied in much of the contemporary literature. Overall, seven factors which attracted great attention considering the influences of economic, social, and geographic contexts can be classified, as shown in, Table 1.

*Urban morphology* refers to the characteristics of the spatial structure and the patterns of the regional economy. It is associated with many environmental topics as well, such as air pollution [20], land use [21], and urban conservation [22]. As for the electricity consumption, Larivière and Lafrance [23] demonstrated that raising the urban density which is defined as inhabitant per km^2^ could decrease the annual city electricity consumption per inhabitant in Canada. Permana et al. [24] confirmed the differences of energy consumption per unit income in three kinds of urban forms in Bandung City which is selected as a typical sample of developing country cities.

*Industrial structure* refers to the number and size distribution of enterprises. It causes significant impacts on the distribution of electricity consumption. Wang et al. [25] decomposed the determinant factors influencing the change trend of industrial electricity consumption in China and characterized these elements. Besides, the economic output, as well as the social and environmental function of different sectors, should be taken into consideration from the systematic perspectives [10]. For instance, Xia et al. [14] introduced the employment variable to evaluate the Chinese industrial sectors. 

*Regulation context* is related to the institution backgrounds of environmental legislation, administration structure, and other settings. Aasen et al. [26] analyzed the influences of the electricity disclosure scheme required by the EU Electricity Directive on Norwegian enterprises and found out that the regulation policy may be inefficient because of the malfunction of program implementation. Chen [27] described the reform process of electricity pricing system proposed by China's central authorities and presented three difficulties of the current promotion for the conservation of energy consumption. The administration powers from the higher authorities are the mainspring of promoting energy efficiency and conservation in China. The regulation of energy consumption relies heavily on mandatory plans or other compulsory measures from above due to lacking of market mechanisms [28].

*Urbanization rate* is measured by the ratio of urban population to the total population. Since the urbanized area does not only include megalopolises, metropolises, but also small cities and towns, urbanization is different from the concentration of population which gives more attention to the central point of the city. For instance, a person who is living in a small town which is far from the central city and undertaking the production of mechanical instruments should be regarded as the urbanization population but not as the people concentrated in the district of the city. Halicioglu [29] found the long-run causality relationship between residential electricity consumption and urbanization in Turkey. Liu [30] studied the linkage between urbanization and energy consumption based on econometric methods including ARDL testing and ECM methods and proposed that urbanization acceleration may harmonize the sustainable development in China. Wiesmann et al. [31] also use the urbanization as an explanation variable to model the residential electricity consumption.

*Price* is bound up with the electricity utilization in terms of the demand and supply of electricity. As the instrument of market adjustment, price scheme together with other factors such as urbanization rate [29] impacts the selection of energy type of the individual and enterprises and eventually determines the amount of electricity consumption. Ziramba [32] estimated the price and income elasticities of residential electricity consumption, showing the price insensitivity of electricity demand in South Africa. Bianco et al. [33] forecasted the nonresidential electricity consumption based on the price analysis using trigonometric grey model.

*Natural condition *is a set of factors which could affect the producing and living conditions and influences the demand function of electricity consumption as the independent variable. For example, the necessity of heating, ventilation, and air condition system in the building absolutely depends on the temperature and humidity. Bessec and Fouquau [34] found the nonlinear relationship between temperature and electricity consumption and distinguished different patterns of this link in European countries by virtue of panel threshold model. Lai et al. [35] modeled the electricity consumption in Macao using mathematic methods and identified the quadratic influence of temperature on electricity consumption. The significance of temperature impacts on electricity was also reported by Larivière and Lafrance [23] and Wiesmann et al. [31]. 

*Resource endowment* is associated with the reserves of mineral, water, and other resources in the region, exerting great influence on the selection and development of industry and indirectly determining the electricity consumption. Burke [36] found that the electricity ladder which is defined by the transition from traditional electricity to renewable power relied heavily on the energy endowments after the investigation of 133 countries.

## 3. Methodology and Data Descriptions

### 3.1. Finite Mixture Model and PCA

 The finite mixture model would be employed to identify the complexity of the electricity consumption in China and scrutinize the influence of the key factors mentioned before. The integration of factor analysis and clustering with category indicators are provided for implementing the policies of electricity administration. Let *y*_*i*_ be the electricity consumption intensity (electricity consumption/GRP) observed for the city *i*, *i* = 1,…, *N*. The sample is assumed to be drawn from a finite mixture model with *K* components. The probability density function for observations is given by

(1) $$\begin{array}{c} f\left(y\;|\;X_{p},X_{c},\Pi ,\Theta ,\alpha \right)=\sum_{l=1}^{K}\Pi_{l}\left(X_{c},\alpha \right)f_{l}\left(y\;|\;\Theta \left(X_{p}\right)\right). \end{array}$$

Here, *X*_*p*_ denotes the predictor vector, *X*_*c*_ is the concomitant variable vector, and *f* (*l* = 1,…, *K*) indicates the component density function with the parameter vector Θ. Π_*l*_ represents the component weight and is defined in the (*K*-1)-dimensional simplex as

(2) $$\begin{array}{c} \sum_{l=1}^{K}\Pi_{l}\left(X_{c},\alpha \right)=1,\;0<\Pi_{l}\left(X_{c},\alpha \right)<1,\forall l, \end{array}$$

where *α* = (*α*_*l*_′)_*l*=1,…,*K*_′ is the parameter vector determining the probability of components. Suppose further that the model is the mixture of Gaussian linear regression, and (1) could be constructed as

(3) ∑l=1KΠl(Xc,α)N(y ∣ μl(Xp),σl2) =∑l=1KΠl(Xc,α)N(y ∣ Xp′βl,σl2).

Here *μ*_*l*_(·) is the mean with linear structure *μ*_*l*_(·) = *X*_*p*_′*β*_*l*_ and *σ*_*l*_^2^ is the variance for the *l*th component normal density  *N*(*y* | *μ*_*l*_(*X*_*p*_), *σ*_*l*_^2^). The coefficients of mixture regression *β*_*l*_ could be *fixed* across all the components, *nested* with some coefficients of components equally, or *varied* between the *K* components. Maximum likelihood (ML) estimation could be used to fit the multivariate Gaussian linear mixture models through the Expectation-Maximization (EM) algorithm [37–40]. In computation practices the component weight Π_*l*_ could be assumed to follow a multinomial logit model [41] as

(4) $$\begin{array}{c} \Pi_{l}\left(X_{c},\alpha \right)=\frac{\exp\left(X_{c}^{\prime }\alpha_{l}\right)}{\sum_{t}\exp\left(X_{c}^{\prime }\alpha_{t}\right)},\;\forall l \end{array}$$

with the constraint *α*_1_ = 0. Information criteria including Akaike Information Criterion (AIC) [42], Bayes Information Criterion (BIC) [43] and Hannan-Quinn Criterion (HQ) [44], and classification criteria including Normalized Entropy Criterion (NEC) [45], Classification Likelihood Criterion (CLC) [46] and Integrated Completed Likelihood-BIC (ICL-BIC) [47] are available for choosing the optimal structure of finite mixture models. 

Principal component analysis, a linear dimension reduction method, is applied to combine the explanatory variables for the finite mixture model. This treatment was inspired by Bai and Ng [48] and Ng and Bai [49] and the analogous technique was also used in Xia et al. [14].

### 3.2. Data Descriptions

#### 3.2.1. Data Definitions

As urban sprawl is generally developed from a specific focal point [50], the district concentration is a main feature of urban morphology. The district under the jurisdiction of the city is regarded as the focal point of the city, especially in China this district is normally the political, economical, and cultural center of the city. The concentrations of GRP (*X*_11_), population (*X*_12_), industrial value added (*X*_13_), fiscal revenue (*X*_14_), and investment in fixed assets (*X*_15_) are selected for measuring *urban morphology* as defined in Table 2.

The shares of industrial output (*X*_21_), industrial employment (*X*_22_), and industrial electricity consumption (*X*_23_) are evaluated by the ratio of industrial value added to GRP, the ratio of industrial employment to total labor population and the weight of industrial electricity consumption in the city respectively. These indicators embody the aspects of economic, social, and environmental impacts and could give a comprehensive description of *industrial structure *(*X*_2_). 

The energy intensity and electricity consumption intensity are the main indicators to be supervised by Chinese energy regulatory system and official statistical bureau. The former one becomes a constraint target in national development programs such as the 12th Five-Year Plan; the latter would be easily monitored in the view of government authorities and also treated as a relatively accurate indicator to reflect the actual conditions of energy consumption due to fewer incentives to manipulate the data on electricity consumption in comparison with those on energy consumption. The decease rates of energy intensity (*X*_31_), energy intensity of industry (*X*_32_), and electricity consumption intensity (*X*_33_) in the province are taken into consideration comprehensively to evaluate the regulation pressure which is exerted by provincial governments on the city authorities for the reflection of *regulation context *(*X*_3_).

The urbanization rate of the province (*X*_41_) is used to capture the provincial heterogeneity in the development due to uneven economic development in China. Since the urbanization process is often closely associated with the transfer of rural surplus labor to urban sectors, the share of nonagricultural population (*X*_42_) is chosen to measure industrial dimension of urbanization. The combination of the urbanization rate of the city (*X*_43_) and the other two variables could overcome the possible statistical bias coming from the incorrect estimated flowing population and gives a comprehensive evaluation of the categorical factor *X*_4_, that is, *urbanization *(*X*_4_). 

Average retail price of electricity which is the synthesis of all kinds of price including residential price, commercial price, and industrial price in China is chosen as an integrative indicator to measure the factor *price* (*X*_5_).

Since temperature is one of the most important factors affecting the electricity consumption as presented in literature, on account of the availability of data, annual average temperature which is recorded in climatological data is selected to measure the characteristics of *natural condition* (*X*_6_).

In view of the dependence of manufacture industry in China's economic growth and the high growth in demand for mineral resource, ensured reserves of iron ore could be proxy for *resource endowment *(*X*_7_).

#### 3.2.2. Data Sources

 The main data sources are China Statistical Yearbook and China City Statistical Yearbook, published by National Bureau of Statistics of China (NBSC). The variables associated with provincial characteristics are taken from NBSC [1] and those associated with city characteristics are abstracted from NBSC [2]. After removing the data with missing explanation variables, a final sample set of 277 cities including 15 cities at subprovince level and 262 cities at prefecture level in 2009 is obtained. The annual average temperature of each city is collected by the authors from public data sources such as the official website of local government. The average retail prices of electricity in the cities are drawn from the report of State Electricity Regulatory Commission [51].

#### 3.2.3. Sample Description

 The descriptive statistics and units of original data are given in Table 3. The great disparity between regions in China exists in consideration of concentration degree. The minimum values of concentration variables are below 10% while the maximum values of these reach to 100%, showing the spatial differences in Chinese urban development. The mean shares of industrial output, employment, and electricity consumption in industry are 50.378%, 45.419%, and 65.964%, respectively, indicating the high weight of electricity consumption in industry even if the output and employment contributions are accounted. Seeing that the average decease rate of industrial energy intensity (8.984%) is smaller than that of total energy intensity (4.908%) at the provincial level during the same period, the energy consumption of industry presents more elasticity than other sectors. The lowest retail price of electricity (298.630 Yuan/MWH) is only about 42.698% of the highest one (699.400 Yuan/MWH) in 2009. Despite the fact that the electricity price is regulated by Chinese government, price mechanism may play certain roles in resource allocations. 

## 4. Results and Discussion

### 4.1. Dimensionality Reduction

 Dimensionality reduction technique could provide crucial variables for understanding the key phenomena of interest without losing the information conveyed by the original data. The results of combined factors via PCA method could be given by

(5) $$\begin{array}{c} X_{1}=0.977X_{11}+0.924X_{12}+0.935X_{13}+0.905X_{14}+0.955X_{15},\\ X_{2}=0.895X_{21}+0.847X_{22}+0.776X_{23},\\ X_{3}=0.493X_{31}+0.841X_{32}+0.927X_{33},\\ X_{4}=0.747X_{41}+0.842X_{42}+0.786X_{43}, \end{array}$$

The selected principle components of urban morphology (*X*_1_), industrial structure (*X*_2_), regulation context (*X*_3_) and urbanization rate (*X*_4_) extracted 88.242%, 70.724%, 60.356%, and 62.840% of squared loading sums, respectively.

### 4.2. Estimation Results

 Considering the commonness of BIC [14, 49] and the robustness of ICL-BIC [47], the two criteria are used for appraising the fitting of mixture models.  Table 4 shows the estimation results for the finite mixture model with best performance of fitting. 

It is notable that the acting structure of factors that impacted on the electricity intensity is distinguishable across the components which are corresponding to the city clusters as shown in Table 5. The factors of urban morphology (*X*_1_), industrial structure (*X*_2_), price (*X*_5_), natural condition (*X*_6_), and resource endowment (*X*_7_) present variance between components, while the influence of regulation pressure (*X*_3_) is fixed and the impact of urbanization degree (*X*_4_) shows a nest structure. Meanwhile, the factor of resource endowment (*X*_7_) is modeled in the finite mixture model as a concomitant variable, indicating this factor influences the electricity intensity indirectly, whereas the other factors as predictors make more direct impacts on the dependent variable.

 The estimations of each factor would be analyzed next. The positive coefficients on urban morphology (X_1_) which is related to the urban concentration degree suggest that promoting the urban concentration would increase the electricity intensity. The moderate concentration of region would bring about economies of scale in the sense of electricity consumption, while the urban sprawling around a central point immoderately would vanish the scale merits and cause diseconomies of scale. The estimated results manifest the excess centralization of urban expansion in China. This centralized trend is most significant in the component 1 with the largest estimated coefficient which is evaluated *cluster 1* of cities. 

The extent of industrial structure (*X*_2_) impacts on electricity intensity is in keeping with that of urban morphology (*X*_1_). That is, as for the electricity intensity, the higher marginal contribution of urban concentration means the greater marginal influence of the rise of industrial weights as viewed from the sizes of estimated coefficients between the categorical factors. It is the evidence that the highly centered development in regions is partially attributed to the significant dependence on industrial output. The combined function of these factors leads to the increasing of electricity intensity and the boost of electricity demand.

The estimated coefficients of −9.677 on regulation context (*X*_3_) suggest that the administrative pressure from the higher level authorities on electricity consumption is the powerful measure to induce decreasing the electricity consumption intensity. Furthermore, as the estimation values are equal between the components, the validity of regulation pressure is identical across the cities owing to the homogeneity of administration system in China. The administration power from top to bottom is still a useful but expedient approach in the context of lacking long-term mechanisms for energy savings.

 Regarding the urbanization rate (*X*_4_), the impacts on electricity consumption intensity presented two levels as the coefficients of 19.305 and 17.365 illustrated. The variables are standardized before estimation, so the impact extents could be compared. The signs of estimates betoken the possibility of long-term increasing of energy consumption intensity due to the urbanization trends in China, but the influences of the factor are inferior to those of urban morphology (*X*_1_) and industrial structure (*X*_2_). 

 The electricity consumption intensity is sensitive to the price of electricity (*X*_5_) in most regions in spite of the fact that the electricity price is regulated and controlled by the government administration. The coefficient of −7.509 on the electricity price (*X*_5_) in component 1 indicates that the enhancement of the electricity price may largely reduce the electricity consumption intensity of the regions in cluster 1. The detailed examination of cluster 1 in Table 5 reveals that most of these cities abound with mineral deposits and rely on resource intensive industry for economic development. This is the evidence of the distortion of electricity price which may induce the discordances of industrial structure such as the overdevelopment of electricity intensive industry. 

As the key variable indicated the natural condition the annual average temperature (*X*_6_) affects the utilization of electricity significantly in the region of clusters 2–4. The values of −0.032, −0.062, and −0.186 on ensured reserves of iron ore (*X*_7_) give the detailed description of the cluster probability which is determined by the concomitant variable. It is revealed by the coefficients that the regions in cluster 1 are most related with the endowment in electricity consumption, followed by the regions in cluster 2, cluster 3, and cluster 4 in order. The industrial structure may be the intermediate link which could explain the indirection of the connection between electricity consumption intensity and the resource endowment. These findings show that the industry distribution near the ore sources is one of the characteristics in China and the influence degrees of the determinants of electricity consumption intensity may be varied with the resource endowment.

## 5. Conclusions and Policy Implications

This study extracts the key factors which determine the electricity consumption intensity of the cities at subprovince and prefecture levels and reveals the acting structure and extent of each determinant which reflects the main characteristics of Chinese social and economic development. The findings of this study are crucial for electricity administration and the specific policies are suggested as follows.

1. Scrutinizing the underlying influences of potential actions before decision making and formulating the comprehensive policy in consideration of regional characteristics may cause better results. The unitary policy may distort the aims of the policymakers and even upset the initial targets because of the vast spread of development degrees and various effects of different factors.
2. Optimizing the spatial layout of regional development would improve the efficiency of electricity consumption and decrease the electricity intensity. Since the GPR, population, industry, public finance, and investment are highly concentrated around the core areas of the cities in China, decentralization of regional planning should be taken into account to upgrade the performance of electricity utilization. It is necessary for the government to balance the economic and social development within the regions.
3. Diversifying the industrial structure, decentralizing economic activities, and establishing multiple cores for regional development would be helpful to achieve lower growth of electricity intensity in China. The integrated consideration of industrial distribution and regional cooperation is needed for the sustainability of economic growth. These immediate actions should be taken especially for the regions in cluster 1 which is most related to the mineral dependent cities.
4. Besides the regulation powers from the administrative systems, the synthetic system with multiple policy instruments should be established. More attention should be paid to the market oriented mechanism. Also, the price system should be given full scope to adjust the relationship between electricity consumption and economic activities.
5. Promoting the urbanization in decentralization way and increasing the employment capacities of industry could relieve the pressure on electricity caused by urbanization in China. The influences of the regional concentrating and industry intensifying are greater than that of the urbanization in development. More attention should be paid on the former factors in order to manage the electricity consumption.
6. Deregulating the electricity price and accelerating the market-oriented reform in energy field could phase down the distortion possibility of energy price system. Extending the industrial chains could lower the electricity consumption in the regions where the development is heavily relied on energy intensive industries. Meanwhile, the unitary energy policy within the country with a vast territory and various degrees of development as China may lead to unrealized results. Energy policies should be synthesized and the multiple factors should be considered adequately before implementation.

## Figures and Tables

**Table 1:** Selected categorical indicators determined electricity consumption in the literature.

Reference	Regions	Contents	Period	Related variables	Categorical indicator
Larivière and Lafrance [23]	Canadian cities	Electricity consumption	1991	Urban density	Urban morphology
				Annual degree-days below 18^°^C	Natural condition
Halicioglu [29]	Turkey	Residential electricity consumption	1968–2005	Real residential electricity price	Price
				Urbanization rate	Urbanization
Bessec and Fouquau [34]	15 European countries	Electricity consumption	1985–2000	Temperature	Natural condition
Lai et al. [35]	Macao	Electricity consumption	2000–2006	Monthly mean temperature	Natural condition
Permana et al. [24]	Bandung city, Indonesia	Household consumption of electricity and liquefied petroleum gas	2007	Urban development forms	Urban morphology
Ziramba [32]	South Africa	Per capita residential electricity consumption	1978–2005	Real residential electricity price	Price
Liu [30]	China	Energy (including electricity) consumption	2009	Urbanization level	Urbanization
Aasen et al. [26]	Norway	Electricity consumption of enterprise	—	Electricity disclosure scheme	Regulation context
Bianco et al. [33]	Romania	Nonresidential electricity consumption	1975–2008	Electricity price	Price
Burke [36]	133 countries	Electricity ladder	1960–2003	Resource reserves	Resource endowment
Wiesmann et al. [31]	Portugal	Residential electricity consumption	2001	Heating/cooling degree-days Urbanization level	Natural condition Urbanization
Chen [27]	China	Energy (mainly electricity) conservation	2004–2011	Differential electricity pricing policy	Regulation context
Wang et al. [25]	China	Change in industrial electricity consumption	1998–2007	Change in industrial structure	Industrial structure

**Table 2:** Description of explanatory variables and category factors.

Variable	Description
*Urban morphology* ^ a^	
GRP concentration	The ratio of Gross Regional Product (GRP) of the district to that of the city
Population concentration	The ratio of population of the district to that of the city
Industrial concentration	The ratio of industrial value added of the district to that of the city
Fiscal concentration	The ratio of government revenue by the district to that by the city
Investment concentration	The ratio of total investment in fixed assets of the district to that of the city
*Industrial structure*	
Share of industrial output	The ratio of industrial value added to GRP in the city
Share of industrial employment	The ratio of the number of employed person in industry to the total of employed person in the city
Share of industrial electricity consumption	The ratio of electricity consumption in local industry to the total of energy consumption in the city
*Regulation context*	
Decease rate of energy intensity	The decease rate of energy consumption per unit of GRP in local province in the corresponding period
Decease rate of energy intensity of industry	The decease rate of energy consumption per unit of industrial value added in local province in the corresponding period
Decease rate of electricity consumption intensity	The decease rate of electricity consumption per unit of GRP in local province in the corresponding period
*Urbanization rate*	
Urbanization degree of the province	The proportion of urban population in the local province
Share of nonagricultural population	The proportion of nonagricultural population in the city
Urbanization degree of the city	The proportion of urban population in the city
*Electricity price*	
Average retail price of electricity	The weighted average of industry, commerce, and residence prices
*Natural condition*	
Annual average temperature	The mean of annual temperature according to urban temperature record in long term
*Resource endowment* ^ b^	
Ensured reserves of iron ore	The amount of iron ore that can be utilized under current conditions in the local province

^a^District refers to the districts under the jurisdiction of the city at subprovince and prefecture levels.

^b^The effects of ensured reserves of coal which had been also taken into account are not significant. The resource endowment of the city may be disturbed by the coal transportation, while the iron ore is often utilized locally in China.

**Table 3:** Descriptive statistics and units of original data.

Variable	Unit	Mean	Standard deviation	Minimum	Maximum
Output concentration (*X*_11_)	%	45.533	23.585	8.038	100.000
Population concentration (*X*_12_)	%	33.224	22.877	4.368	100.000
Industrial concentration (*X*_13_)	%	48.899	25.140	5.447	100.000
Fiscal concentration (*X*_14_)	%	52.600	25.490	5.931	100.000
Investment concentration (*X*_15_)	%	45.047	23.766	7.300	100.000
Share of industrial output (*X*_21_)	%	50.378	11.814	9.740	82.390
Share of industrial employment (*X*_22_)	%	45.419	15.178	6.550	79.450
Share of industrial electricity consumption (*X*_23_)	%	65.964	18.926	7.243	98.839
Decease rate of energy intensity (*X*_31_)	%	4.908	2.295	0.830	9.730
Decease rate of energy intensity of industry (*X*_32_)	%	8.984	3.057	0.030	15.100
Decease rate of electricity consumption intensity (*X*_33_)	%	5.330	0.812	2.810	6.970
Urbanization degree of the province (*X*_41_)	%	46.903	8.743	29.890	63.400
Share of nonagricultural population (*X*_42_)	%	61.423	25.211	13.480	100.000
Urbanization degree of the city (*X*_43_)	%	44.321	14.790	13.840	100.000
Average retail price of electricity (*X*_5_)	Yuan/MWH	512.013	85.014	298.630	699.400
Annual average temperature (*X*_6_)	^ °^C	14.653	5.294	−1.880	25.500
Ensured reserves of iron ore (*X*_7_)	100 million tons	9.916	16.698	0.000	70.200

Note. Some maximum values of concentration variables are 100% because of the fulfillment of urbanization in some region.

**Table 4:** Estimation results and variable structure for the finite mixture model^a^.

Variable	Structure	Coefficient estimates			
		Component 1	Component 2	Component 3	Component 4
		Predictor			
Intercept	Varied	5461.407^∗∗∗^ (919.115)	379.656^∗∗∗^ (87.482)	1774.662^∗∗∗^ (158.055)	982.128^∗∗∗^ (61.198)
*X*_1_	Varied	113.915^∗∗∗^ (40.749)	23.923^∗∗∗^ (2.821)	59.830^∗∗∗^ (6.378)	40.738^∗∗∗^ (3.021)
*X*_2_	Varied	66.189 (92.219)	22.124^∗∗∗^ (6.895)	48.875^∗∗∗^ (12.326)	32.844^∗∗∗^ (4.232)
*X*_3_	Fixed	−9.677^∗∗^ (4.896)	−9.677^∗∗^ (4.896)	−9.677^∗∗^ (4.896)	−9.677^∗∗^ (4.896)
*X*_4_	Nested	19.305^∗∗∗^ (7.366)	19.305^∗∗∗^ (7.366)	17.365^∗∗^ (6.991)	17.365^∗∗^ (6.991)
*X*_5_	Varied	−7.509^∗∗∗^ (1.315)	−0.287 (0.185)	−2.910^∗∗∗^ (0.414)	−0.759^∗∗∗^ (0.130)
*X*_6_	Varied	−52.716 (35.108)	6.815^∗∗^ (2.750)	25.498^∗∗∗^ (7.160)	−7.531^∗∗∗^ (1.774)

Concomitant variable					
Intercept	Varied	—	2.187^∗∗∗^ (0.441)	1.976^∗∗∗^ (0.473)	2.285^∗∗∗^ (0.516)
*X*_7_	Varied	—	−0.032^∗∗∗^ (0.012)	−0.062^∗∗^ (0.024)	−0.186^∗∗^ (0.092)

^a^The standard errors of coefficient estimates are in parentheses. ^∗∗^ and ^∗∗∗^ denote significance at 5% and 1% levels, respectively. All the explanatory variables are standardized.

**Table 5:** Clustering membership of the finite mixture model.

*Cluster 1*	
Wuhai (Inner Mongolia), Anshan (Liaoning), Fushun (Liaoning), Benxi (Liaoning), Yingkou (Liaoning), Fuxin (Liaoning), Liaoyang (Liaoning), Huludao (Liaoning), Laiwu (Shandong), Jiaozuo (Henan), Panzhihua (Sichuan), Qujing (Guizhou), Tongchuan (Shaanxi), Jiayuguan (Gansu), Baiyin (Gansu), Shizuishan (Ningxia), Zhongwei (Ningxia)	
*Cluster 2*	
Shijiazhuang (Hebei), Handan (Hebei), Baoding (Hebei), Cangzhou (Hebei), Langfang (Hebei), Hengshui (Hebei), Jincheng (Shanxi), Shuozhou (Shanxi), Luliang (Shanxi), Hohhot (Inner Mongolia), Erdos (Inner Mongolia), Hulunbuir (Inner Mongolia), Bayannur (Inner Mongolia), Ulanqab (Inner Mongolia), Shenyang (Liaoning), Dalian (Liaoning), Dandong (Liaoning), Jinzhou (Liaoning), Panjin (Liaoning), Tieling (Liaoning), Chaoyang (Liaoning), Changchun (Jilin), Liaoyuan (Jilin), Baishan (Jilin), Songyuan (Jilin), Baicheng (Jilin), Harbin (Heilongjiang), Jiamusi (Heilongjiang), Wuxi (Jiangsu), Suzhou (Jiangsu), Nantong (Jiangsu), Lianyungang (Jiangsu), Yancheng (Jiangsu), Taizhou (Jiangsu), Suqian (Jiangsu), Zhoushan (Zhejiang), Hefei (Anhui), Wuhu (Anhui), Huangshan (Anhui), Chuzhou (Anhui), Bozhou (Anhui), Xuancheng (Anhui), Fuzhou (Fujian), Xiamen (Fujian), Putian (Fujian), Quanzhou (Fujian), Longyan (Fujian), Ningde (Fujian), Nanchang (Jiangxi), Jingdezhen (Jiangxi), Fuzhou (Jiangxi), Jinan (Shandong), Qingdao (Shandong), Zaozhuang (Shandong), Dongying (Shandong), Yantai (Shandong), Weifang (Shandong), Taian (Shandong), Weihai (Shandong), Dezhou (Shandong), Liaocheng (Shandong), Xuchang (Henan), Luohe (Henan), Zhoukou (Henan), Xiangfan (Hubei), Xiaogan (Hubei), Changsha (Hunan), Changde (Hunan), Yiyang (Hunan), Guangzhou (Guangdong), Heyuan (Guangdong), Yangjiang (Guangdong), Jieyang (Guangdong), Beihai (Guangxi), Fangchenggang (Guangxi), Yulin (Guangxi), Chengdu (Sichuan), Zigong (Sichuan), Luzhou (Sichuan), Deyang (Sichuan), Mianyang (Sichuan), Suining (Sichuan), Neijiang (Sichuan), Nanchong (Sichuan), Meishan (Sichuan), Yibin (Sichuan), Guangan (Sichuan), Dazhou (Sichuan), Bazhong (Sichuan), Ziyang (Sichuan), Yuxi (Guizhou), Baoshan (Guizhou), Lincang (Guizhou), Xi'an (Shaanxi), Baoji (Shaanxi), Xianyang (Shaanxi), Weinan (Shaanxi), Yan'an (Shaanxi), Hanzhong (Shaanxi), Yulin (Shaanxi), Shangluo (Shaanxi), Jiuquan (Gansu), Qingyang (Gansu)	
*Cluster 3*	
Tangshan (Hebei), Qinhuangdao (Hebei), Xingtai (Hebei), Zhangjiakou (Hebei), Chengde (Hebei), Yangquan (Shanxi), Yuncheng (Shanxi), Hegang (Heilongjiang), Daqing (Heilongjiang), Quzhou (Zhejiang), Maanshan (Anhui), Tongling (Anhui), Xinyu (Jiangxi), Zibo (Shandong), Rizhao (Shandong), Zhengzhou (Henan), Luoyang (Henan), Anyang (Henan), Shangqiu (Henan), Huangshi (Hubei), Ezhou (Hubei), Xiangtan (Hunan), Zhangjiajie (Hunan), Huaihua (Hunan), Shaoguan (Guangdong), Shantou (Guangdong), Dongguan (Guangdong), ZhongShan (Guangdong), Guigang (Guangxi), Baise (Guangxi), Hezhou (Guangxi), Laibin (Guangxi), Guangyuan (Sichuan), Leshan (Sichuan), Yaan (Sichuan), Guiyang (Guizhou), Zunyi (Guizhou), Anshun (Guizhou), Lanzhou (Gansu), Zhangye (Gansu), Pingliang (Gansu), Xining (Qinghai), Wuzhong (Ningxia)	
*Cluster 4*	
Taiyuan (Shanxi), Datong (Shanxi), Changzhi (Shanxi), Jinzhong (Shanxi), Xinzhou (Shanxi), Linfen (Shanxi), Baotou (Inner Mongolia), Chifeng (Inner Mongolia), Tongliao (Inner Mongolia), Jilin (Jilin), Siping (Jilin), Tonghua (Jilin), Qiqihar (Heilongjiang), Jixi (Heilongjiang), Shuangyashan (Heilongjiang), Yichun (Heilongjiang), Qitaihe (Heilongjiang), Mudanjiang (Heilongjiang), Heihe (Heilongjiang), Suihua (Heilongjiang), Nanjing (Jiangsu), Xuzhou (Jiangsu), Changzhou (Jiangsu), Huaian (Jiangsu), Yangzhou (Jiangsu), Zhenjiang (Jiangsu), Hangzhou (Zhejiang), Ningbo (Zhejiang), Wenzhou (Zhejiang), Jiaxing (Zhejiang), Huzhou (Zhejiang), Shaoxing (Zhejiang), Jinhua (Zhejiang), Taizhou (Zhejiang), Lishui (Zhejiang), Bengbu (Anhui), Huainan (Anhui), Huaibei (Anhui), Anqing (Anhui), Fuyang (Anhui), Suzhou (Anhui), Chaohu (Anhui), Liuan (Anhui), Chizhou (Anhui), Sanming (Fujian), Zhangzhou (Fujian), Nanping (Fujian), Pingxiang (Jiangxi), Jiujiang (Jiangxi), Yingtan (Jiangxi), Ganzhou (Jiangxi), Jian (Jiangxi), Yichun (Jiangxi), Shangrao (Jiangxi), Jining (Shandong), Linyi (Shandong), Binzhou (Shandong), Heze (Shandong), Kaifeng (Henan), Pingdingshan (Henan), Hebi (Henan), Xinxiang (Henan), Puyang (Henan), Sanmenxia (Henan), Nanyang (Henan), Xinyang (Henan), Zhumadian (Henan), Wuhan (Hubei), Shiyan (Hubei), Yichang (Hubei), Jingmen (Hubei), Jingzhou (Hubei), Huanggang (Hubei), Xianning (Hubei), Suizhou (Hubei), Zhuzhou (Hunan), Hengyang (Hunan), Shaoyang (Hunan), Yueyang (Hunan), Chenzhou (Hunan), Yongzhou (Hunan), Loudi (Hunan), Shenzhen (Guangdong), Zhuhai (Guangdong), Foshan (Guangdong), Jiangmen (Guangdong), Zhanjiang (Guangdong), Maoming (Guangdong), Zhaoqing (Guangdong), Huizhou (Guangdong), Shanwei (Guangdong), Qingyuan (Guangdong), Chaozhou (Guangdong), Nanning (Guangxi), Liuzhou (Guangxi), Guilin (Guangxi), Wuzhou (Guangxi), Qinzhou (Guangxi), Hechi (Guangxi), Chongzuo (Guangxi), Haikou (Hainan), Sanya (Hainan), Liupanshui (Guizhou), Kunming (Guizhou), Zhaotong (Guizhou), Lijiang (Guizhou), Puer (Guizhou), Ankang (Shaanxi), Tianshui (Gansu), Wuwei (Gansu), Dingxi (Gansu), Longnan (Gansu), Yinchuan (Ningxia), Guyuan (Ningxia)	

## Appendix

See Table 5.
